# 晚期肺鳞癌患者肿瘤标志物测定的临床分析

**DOI:** 10.3779/j.issn.1009-3419.2016.10.01

**Published:** 2016-10-20

**Authors:** 平 梁, 峻岭 李

**Affiliations:** 1 100021 北京，国家癌症中心/中国医学科学院北京协和医学院肿瘤医院内科 Department of Medical Oncology, National Cancer Center/Cancer Hospital, Chinese Academy of Medical Sciences and Peking Union Medical College, Beijing 100021, China; 2 100122 北京，北京市朝阳区三环肿瘤医院内科 Department of Medical Oncology, San Huan Cancer Hospital in Chao Yang, Beijing 100122, China

**Keywords:** 肺鳞癌, 临床特征, 肿瘤标志物, 阳性率, Squamous cell lung cancer, Clinical characteristics, Tumor markers, Sensitivity

## Abstract

**背景与目的:**

肺鳞癌由于发病隐匿，早期无明显症状，往往到晚期才得以诊断。本研究旨在描述性分析晚期肺鳞癌患者的基本特征和多种肿瘤标志物检测水平及阳性率情况，评价其临床价值。

**方法:**

以2011年1月-2015年12月期间于中国医学科学院肿瘤医院诊治的晚期肺鳞癌患者作为研究对象，通过病历回顾收集相关资料，描述性分析晚期肺鳞癌患者基本特征、肿瘤标志物检测水平和阳性率。

**结果:**

260例患者的平均年龄为（59.4±9.2）岁，男性223例（85.8%），女性37例（14.2%）。203例（78.1%）有吸烟史，8例（3.1%）有癌症家族史。细胞角质蛋白19的片断（cytokerantin 19 fragment, CYFRA21-1）的检测阳性率最高（71.2%）。不同肿瘤原发灶（tumor, T）分期和淋巴结受累（node, N）分期患者五种指标检测水平无统计学差异（*P*＞0.05），仅鳞状细胞癌相关抗原（squamous cell carcinoma antigen, SCC）在不同T分期的检测阳性率有统计学差异（*P*=0.035）。二联阳性率最高的为CYFRA21-1+蛋白质类的癌胚抗原（carcinogen-embryonic antigen, CEA），阳性率为82.7%，三联阳性率最高的为癌抗原12-5（cancer antigen 125, CA125）+CYFRA21-1+CEA，阳性率为84.6%，四联阳性率最高为CA125+CYFRA21-1+CEA+酶类标志物神经烯醇化酶（neuron specific enolase, NSE），阳性率为85.0%，五联的阳性率为86.2%。

**结论:**

CYFRA21-1的检测阳性率最高，单项肿瘤标志物的检测灵敏度较低，联合检测可提高对肺鳞癌的诊断灵敏度，首选CA125、CYFRA21-1和CEA联合。

## Abstract (en)

**Background and objective:**

Due to it's concealment and no obvious symptoms, lung squamous carcimoma often has advanced disease when diagnosed. The aims of this study were to describe the characteristics of the disease, to evaluate the clinical importance of detection of multiple tumor markers in patients with squamous cell carcinoma of the lung.

**Methods:**

The characteristics of all patients with advanced squamous cell lung cancer treated in Beijing Cancer Hospital of Chinese Academy of Medical Sciences during Jan. 2011 to Dec. 2015 were identified by cases reviewing and data extracting. The characteristics, detection levels and sensitivity of multiple tumor makers among patients were described.

**Results:**

The 260 patients were treated with mean age of (59.4±9.2) years, 85.8% (*n*=223) of them were male, 14.2% (*n*=37) of them were female. 78.1% (*n*=203) of all were smokers and 3.1% (*n*=8) of patients had family history of tumor. The positive rate of cytokerantin 19 fragment (CYFRA21-1) was 71.2%, which was the highest among five tumor markers. The five tumor markers median level had no statistical significance between different tumor (T) stages and node (N) stages (all *P* > 0.05), only the positive rate of SCC had statistical significance between different T stages (*P*=0.035). The combination measurement of CYFRA21-1+carcinogen-embryonic antigen (CEA), CYFRA21-1+CEA+cancer antigen (CA125), CA125+CYFRA21-1+CEA+neuron specific enolase (NSE), and CA125+CYFRA21-1+NSE+CEA+squamous cell carcinoma antigen (SCC) were better and had higher clinical values, the positive rates were 82.7%, 84.6%, 85.0% and 86.2%, respectively.

**Conclusion:**

The positive rate of CYFRA21-1 was the highest and the sensitivity of single test of five tumor markers was low, the combination of multiple tumor markers increased the sensitivity of diagnosis of SQCLC, the combination of CA125, CYFRA21-1 and CEA was the best choice.

肺癌是我国最常见的恶性肿瘤之一，发病率和病死率居各种恶性肿瘤之首^[1]^。非小细胞肺癌是肺癌的主要类型，占所有肺癌的80%左右^[2]^，而其中肺鳞癌的发病约占了非小细胞肺癌的25%^[3]^。由于肺鳞癌早期病情较为隐匿，确诊时已进展到晚期，错过手术治疗的最佳时机，化疗成为晚期肺鳞癌治疗的主要手段^[4]^。因此，对肺鳞癌患者早期诊断及后期治疗疗效的评价具有重要意义。肿瘤标志物在肿瘤普查、诊断、判断预后和转归、评价疗效等方面具有较大实用价值^[5]^。目前，蛋白质类的癌胚抗原（carcinogen-embryonic antigen, CEA）、癌抗原12-5（cancer antigen, CA125）、细胞角质蛋白19的片断（cytokerantin 19 fragment, CYFRA21-1）以及酶类标志物神经烯醇化酶（neuron specific enolase, NSE）是较常用的标志物^[6]^。另外，有研究^[7]^表明鳞状细胞癌相关抗原（squamous cell carcinoma antigen, SCC）和CYFRA21-1对肺鳞癌患者有重要检测价值。本研究旨在分析2011年1月-2015年12月中国医学科学院肿瘤医院收治的260例晚期肺鳞癌患者的CEA、CA125、CY21-1、NSE和SCC水平，并对患者基本特征进行描述性分析。

## 1 材料与方法

### 1.1 患者资料

中国医学科学院肿瘤医院2011年1月-2015年12月期间，初诊经病理、影像学检查确诊为Ⅳ期肺鳞癌患者共260例，男性223例，女性37例，年龄37岁-83岁。根据国际抗癌联盟（Union for International Cancer Control, UICC）1997年修订及颁布的肿瘤-淋巴结-转移（tumor node metastasis, TNM）分期法分期，通过病历回顾对相关资料进行收集。

### 1.2 分析内容

对患者社会人口学特征（年龄、性别、职业类型）、发病相关资料（吸烟、戒烟、家族病史情况）和临床特点（发病部位、肿瘤分化程度、首发症状、临床分期等）、肿瘤标志物检测值及检测阳性率进行回顾性描述分析，并对比不同肿瘤原发灶分期和不同淋巴结转移情况的患者肿瘤标志物检测值和检测阳性率差异，以及不同肿瘤标志物联合检测的阳性率差异。

### 1.3 检测指标

260例患者均测定了CEA、CA125、CYFRA21-1、SCC和NSE水平，CEA、CA125、CYFRA21-1和NSE采用光化学发光法/罗氏测定，SCC采用酶免法/康乃格测定，参考值范围分别是CEA为0-5 μg/L；CA125为0-35 μ/mL；CYFRA21-1为0-3.3 μg/L；SCC为0-1.5 μg/L；NSE为0-18 μg/L。

### 1.4 统计学方法

将研究所得数据录入Stata 12.0软件中进行统计学分析。计量资料采用均数±标准差（Mean±SD）或中位数以及极值（最大值、最小值）表示，非正态分布的数据多组间比较采用*Kruskal*-*wallis*检验；计数资料使用频数（*n*）和百分比（%）表示，组间比较使用χ^2^检验或*Fisher*确切检验。*P*＜0.05为差异有统计学意义。

## 2 结果

### 2.1 患者基本特征

260例肺鳞癌患者中，男性223例（85.8%），女性37例（14.2%），平均年龄（59.4±9.2）岁（表 1）。其中，病变部位为右肺146例（上叶67例、中叶21例、下叶46例、其他12例）；左肺114例（上叶70例、下叶39例、其他5例）。203例（78.1%）患者有吸烟史，其中，吸烟指数≥400年支的患者占吸烟人数的87.2%，吸烟者平均的吸烟史长达（33.6±10.3）年，初诊时仅有50例（占吸烟人数的24.6%）患者已戒烟。8例（3.1%）患者有癌症家族史。230例（88.5%）患者出现不适症状后才就诊，主要症状包括：咳嗽（164例）、咳痰（101例）、气短（54例）和胸背痛（49例）、痰中带血（41例）。无症状患者30例（11.5%），在常规体检过程中得以诊断和发现。

**1:** 肺鳞癌患者的基本特征 The demographic characters of the study subjects

Item	Mean/*n*	SD/Proportion
Age (yr)	59.4	9.2
Original tumor site		
Right lung	146	56.2%
Left lung	114	43.8%
Smoking status		
Never	57	21.9%
≤200 cigarettes per year	9	4.4%
200-400 cigarettes per year	17	8.4%
≥400 cigarettes per year	177	87.2%
Smoking history/year	33.6	10.3
Family history of tumor	8	3.1%
Stage (T^a^)		
T_4_	56	21.5%
T_3_	27	10.4%
T_2_	125	48.1%
T_1_	12	4.6%
Indeterminate	40	15.4%
Regional lymph node (N^b^)		
N_3_	91	35.0%
N_2_	107	41.2%
N_1_	11	4.2%
N_0_	7	2.7%
Indeterminate	44	16.9%
^a^: clinical stages were categorized by original tumor, with the increase of tumor size and the increase of the adjacent tissue affected scope, showed in turn by T1-T4; ^b^: clinical stages were categorized by regional lymph nodemetastasis, lymph node with no cell metastasis was showed by N0, with the increase of the degree and scope of lymph node metastasis, showed in turn by N1-N3.		

美国东部肿瘤协作组（Eastern Cooperative Oncology Group, ECOG）体能状态评分为0分-1分有251例（96.6%），≥2分有9例（3.4%）。46例（17.7%）患者发生脑部转移，其中28例单发转移，18例多发转移。根据肿瘤分化程度分层，中分化为30例，低分化为84例，无法判断分化情况的患者146例。根据肿瘤原发灶体积和邻近组织受累范围的情况对患者进行临床分期，有40例患者（15.4%）不确定分期；确定分期的患者中，T2期患者占比最高，为48.1%（*n*=125），其次是T4期的21.5%（*n*=56）。根据区域淋巴结受累程度对患者进行分期，淋巴结未受累患者7例（2.7%）、N1期11例（4.2%）、N2期107例（41.2%）、N3期91例（35.0%），还有44例（16.9%）淋巴结转移不确定。

### 2.2 患者总体指标检测结果

260例患者所检测的五个指标结果显示（表 2），检测值分布呈偏态分布，CA125、CYFRA21-1、NSE、SCC、CEA的中位数分别是23.4 U/mL、6.9 μg/L、15.1 μg/L、1.3 μg/L、3.8 μg/L，CA125、NSE、SCC和CEA的检测阳性率均在50%以下，CYFRA21-1的检测阳性率最高为71.2%，其次为SCC，检测阳性率为41.5%。

**2:** 患者检测值分布及检测阳性率 Results of tumor marker determination and sensitivity of all patients

Item	CA125 (U/mL)	CYFRA21-1 (*μ*g/L)	NSE (*μ*g/L)	SCC (*μ*g/L)	CEA (*μ*g/L)
Range	3.3-1, 000.0	0.01-403.0	0.7-146.1	0.2-70.0	0.2-572.1
Median	23.4	6.9	15.1	1.3	3.8
Sensitivity	37.3%	71.2%	26.2%	41.5%	36.5%
CA125: cancer antigen 125; CYFRA21-1: cytokerantin 19 fragment; NSE: neuron specific enolase; SCC: squamous cell carcinoma antigen; CEA: carcinogen-embryonic antigen.					

### 2.3 不同肿瘤原发灶分期患者肿瘤标志物水平比较

将患者按照肿瘤原发灶情况不同的分期进行分析，CA125的中位水平随着分期的增高有增加的趋势（表 3）。不同分期患者关于肿瘤标志物水平差异的非参数检验结果显示，不同分期之间的CEA、CA125、CYFRA21-1、SCC及NSE水平的比较均无统计学差异（*P*＞0.05）。

**3:** 不同临床分期患者肿瘤标志物检测水平比较 Comparison of tumor marker level in serum between different stages of tumor

Item	CA125 (U/mL)	CYFRA21-1 (*μ*g/L)	NSE (*μ*g/L)	SCC (*μ*g/L)	CEA (*μ*g/L)
	Median (Range)	Median (Range)	Median (Range)	Median (Range)	Median (Range)
T_4_	37.7 (7.0, 504.0)	7.0 (1.4, 121.0)	16.0 (1.4, 44.9)	1.6 (0.3, 70.0)	3.5 (0.7, 113.0)
T_3_	24.5 (5.1, 147.0)	7.5 (1.4, 91.9)	14.0 (2.2, 73.7)	2.0 (0.4, 69.0)	3.7 (1.1, 26.7)
T_2_	20.6 (3.3, 1, 000.0)	5.4 (0.01, 103.4)	14.6 (0.7, 146.1)	1.2 (0.3, 70.0)	4.2 (0.8, 337.0)
T_1_	20.5 (7.0, 212.2)	4.1 (1.1, 23.1)	15.6 (7.1, 25.5)	1.7 (0.5, 11.3)	2.8 (0.2, 31.0)
*χ*^2^ value	5.3	6.239	2.482	6.189	6.397
*P* value	0.151, 1	0.100, 6	0.478, 5	0.102, 8	0.093, 8
Clinical stages were categorized by original tumor, with the increase of tumor size and the increase of the adjacent tissue affected scope, showed in turn by T1-T4.					

### 2.4 不同区域淋巴结转移患者肿瘤标志物水平比较

将患者按照区域淋巴结受累程度不同的分期进行分析，CEA的中位水平随分期增高有增加的趋势，NSE和SCC的淋巴结未受累患者的中位水平最高（表 4）。淋巴结未受累患者、N1期、N2期、N3期患者关于肿瘤标志物中位水平差异的非参数检验结果显示，不同分期之间的CEA、CA125、CYFRA21-1、SCC及NSE水平的比较均无统计学差异（*P*＞0.05）。

**4:** 区域淋巴结转移患者肿瘤标志物检测水平比较 Comparison of tumor marker level in serum between patients with different transition of regional lymph node

Item	CA125 (U/mL)	CYFRA21-1 (*μ*g/L)	NSE (*μ*g/L)	SCC (*μ*g/L)	CEA (*μ*g/L)
	Median (Range)	Median (Range)	Median (Range)	Median (Range)	Median (Range)
N_3_	27.0 (6.6, 611.5)	8.3 (1.5, 403.0)	15.6 (0.7, 146.1)	1.2 (0.3, 70.0)	4.2 (0.2, 572.1)
N_2_	25.2 (3.3, 1, 000.0)	6.3 (0.01, 121.0)	15.1 (1.5, 119.3)	1.3 (0.2, 70.0)	3.9 (0.5, 138.0)
N_1_	35.1 (9.6, 96.4)	6.7 (1.35, 60.9)	13.1 (5.9, 44.9)	1.5 (0.3, 11.3)	3.7 (1.7, 31.0)
N_0_	16.0 (7.3, 84.5)	6.9 (2.9, 12.8)	16.2 (6.6, 19.7)	2.9 (0.6, 19.6)	3.2 (1.7, 8.6)
*χ*^2^ value	1.964	1.831	1.604	2.226	1.105
*P* value	0.580	0.608	0.659	0.527	0.776
Clinical stages were categorized by regional lymph nodemetastasis, lymph node with no cell metastasis was showed by N0, with the increase of the degree and scope of lymph node metastasis, showed in turn by N1-N3.					

### 2.5 不同肿瘤病发灶分期和不同区域淋巴结转移患者肿瘤标志物阳性率比较

将患者按照肿瘤原发灶情况不同的分期中，CA125和CYFRA21-1在不同分期患者中的阳性率随分期增高有增加的趋势，但各分期无统计学差异。五个指标中CYFRA21-1的阳性率最高，各分期的阳性率均高于50%。五个指标中仅SCC在各分期的阳性率有统计学差异（*P*=0.035）。将患者按照区域淋巴结受累程度不同的分期中，CYFRA21-1在不同分期患者中的阳性率均高于50%，CYFRA21-1和NSE的阳性率随分期的增高有增加的趋势，但五个指标在各分期的阳性率均无统计学差异（*P*＞0.05）（表 5）。

**5:** 不同临床分期和不同区域淋巴结转移患者肿瘤标志物的阳性检测率比较 Comparison of sensitivity of tumor markers between patients with different stages of tumor and different transition of regional lymph node

Item	CA125	CYFRA21-1	NSE	SCC	CEA
Stage					
T_4_	50.0%	78.6%	33.9%	48.2%	26.8%
T_3_	37.0%	77.8%	18.5%	51.9%	29.6%
T_2_	31.2%	67.2%	22.4%	30.4%	41.6%
T_1_	16.7%	58.3%	25.0%	50.0%	25.0%
*P* value	0.050	0.263	0.338	0.035	0.199
Regional lymph node					
N_3_	38.5%	75.8%	29.7%	34.1%	37.4%
N_2_	39.3%	72.9%	28.0%	44.9%	38.3%
N_1_	45.5%	63.6%	18.2%	36.4%	36.4%
N_0_	14.3%	71.4%	14.3%	57.1%	28.6%
*P* value	0.611	0.794	0.841	0.333	0.992
Clinical stages were categorized by original tumor, with the increase of tumor size and the increase of the adjacent tissue affected scope, showed in turn by T1-T4; Clinical stages were categorized by regional lymph nodemetastasis, lymph node with no cell metastasis was showed by N0, with the increase of the degree and scope of lymph node metastasis, showed in turn by N1-N3.					

### 2.6 不同肿瘤标志物联合检测的阳性率比较

将不同肿瘤标志物联合检测的阳性率结果显示，随着联合肿瘤标志物个数的增加，联合检测的阳性率有升高的趋势，其中将CEA、CA125、CYFRA21-1、SCC和NSE五指标联合的阳性率最高为86.2%（表 6）。

**6:** 不同肿瘤标志物联合检测的阳性率比较 Comparison of positive rate of different combined tumor markers

Group	Sensitivity
Two-combined	
CA125+CYFRA21-1	81.5%
CA125+NSE	58.8%
CA125+SCC	67.3%
CA125+CEA	64.2%
CYFRA21-1+NSE	77.7%
CYFRA21-1+SCC	78.5%
CYFRA21-1+CEA	82.7%
NSE+SCC	58.1%
NSE+CEA	54.6%
SCC+CEA	62.7%
Three-combined	
CA125+CYFRA21-1+NSE	82.3%
CA125+CYFRA21-1+SCC	82.7%
CA125+CYFRA21-1+CEA	84.6%
CA125+NSE+SCC	73.5%
CA125+NSE+CEA	69.6%
CA125+SCC+CEA	75.0%
CYFRA21-1+NSE+SCC	79.6%
CYFRA21-1+NSE+CEA	83.1%
CYFRA21-1+SCC+CEA	84.2%
NSE+SCC+CEA	69.2%
More-combined	
CA125+CYFRA21-1+NSE+SCC	83.5%
CA125+CYFRA21-1+NSE+CEA	85.0%
CA125+NSE+SCC+CEA	84.6%
CYFRA21-1+NSE+SCC+CEA	78.5%
CA125+CYFRA21-1+NSE+SCC+CEA	86.2%

## 3 讨论

肺鳞癌已成为威胁吸烟者健康方面的一大难题，肺鳞癌多与吸烟密切相关^[8]^。有研究^[9]^发现，在肺鳞癌和小细胞癌患者中吸烟率达到75%以上，明显高于其他病理类型。本组病历中，78.1%患者有吸烟史，吸烟指数≥400年支的患者占吸烟人数的87.2%，平均的吸烟史长达33年。其中男性吸烟患者共191例，占男性人数的85.7%；女性吸烟患者共12例，占女性人数的32.4%，吸烟可能是男性发病率高于女性的主要原因，本组分析中男性患者约为女性的6倍。因此，人们应提高对肺癌的认识，政府应当加大对疾病的宣传，了解吸烟的危害，远离危害健康的隐患。另外，癌症家族史与肺癌的发生有密切关系^[10]^，本组患者中3.1%的患者其直系亲属有癌症家族史。

肺鳞癌作为肺癌的重要类型，不同于其他亚型，其在60岁以下人群中发病率较高，由于年轻人群对疾病具有较强的抵抗能力，加之肺鳞癌早期发病隐匿，多数患者确诊时已是晚期^[11]^。本研究中50.8%的患者年龄小于60岁，总人群中88.5%的患者是因症状出现以后才就诊的，这一结果与Peng等^[12]^报道结果一致。患者得以诊断时已达晚期，其主要原因可能是通过普查或体检发现的病例较少，本研究肺鳞癌患者中，仅11.5%的患者是通过常规体检得以诊断，这一结果进一步说明目前的肺鳞癌患者早期诊断率低，患者对于自身的健康管理意识还有待提高，尤其是年轻人也应定期进行健康检查。本组肺鳞癌发病部位右侧多于左侧（146:114），上叶多于下叶（右侧67:46，左侧70:39），与Cui等^[13]^研究相似，发病部位的差异具体原因还需要进一步研究。

血清肿瘤标志物的主要临床应用价值在于对已确诊的肿瘤患者治疗疗效的判断以及复发监测，好的标志物应当是特异性、敏感性都高，并且能反应病情变化^[6]^。肺癌的血清肿瘤标志物因缺乏敏感性特异性，并不被美国国家综合癌症网（National Comprehensive Cancer Network, NCCN）肺癌诊疗指南^[14]^推荐作为单独评估临床疗效的依据。本研究结果显示，本组患者五种肿瘤标志物的分布均不是正态分布，CA125、CYFRA21-1、NSE、SCC、CEA的中位数分别是23.4 U/mL、6.9 μg/L、15.1 μg/L、1.3 μg/L、3.8 μg/L，而且每个指标都有患者超出所能测试的极值。分期后的分布结果显示，按照肿瘤原发灶情况不同的分期，CA125的中位水平随分期增高有增加的趋势，但各分期无统计学差异，其他四种指标在各分期患者中无明显变化趋势。按照区域淋巴结受累程度不同的分期，CEA的中位水平随分期增高有增加的趋势，但各分期均无统计学差异。肺鳞癌患者CYFRA21-1阳性率在诊断时的T分期与N分期有相同的变化趋势，提示其与预后相关，与Zhang等^[15]^的研究一致。

血清肿瘤标志物对肺癌的诊断虽有重要价值，但单一的标志物检测阳性率过低，本研究结果显示，肺鳞癌患者的五个检测指标中CYFRA21-1的检测阳性率最高为71.2%，与国内外其他研究^[7, 16]^结果（＞70%）基本一致。其次为SCC阳性率41.5%，而NSE的检测阳性率仅为26.2%。这一结果也进一步验证了Chen等^[17]^和Wang等^[18]^的研究结果，CYFRA21-1是检测肺鳞癌的首选肿瘤标志物。另外，CYFRA21-1与患者诊断时的T分期和N分期有关，随着分期的增高阳性率有增加的趋势。虽然单一肿瘤标志物检测对肺癌诊断有一定的帮助，但其敏感度难以满足临床对肺癌早期诊断、鉴别诊断、疗效评定及预后评估的要求，因此联合检测相关标志物在肺癌鉴别诊断中尤为必要。为提高诊断灵敏度，对这五个指标做联合平行试验时，具有互补性的肿瘤标志物联合检测可以提高对肺鳞癌的敏感度。二联阳性率最高的为CYFRA21-1+CEA（82.7%），三联阳性率最高的为CA125+CYFRA21-1+CEA（84.6%），四联阳性率最高为CA125+CYFRA21-1+NSE+CEA（85.0%），其中，五联的阳性率最高，为86.2%。本研究多项联合检测的阳性率均高于Zhang等^[15]^的报道。由于项目联合越多，假阳性越高，所需费用也越高^[15]^，因此对于肺鳞癌患者建议临床采用CA125+CYFRA21-1+CEA三联平行检测。
